# An enhanced individual placement and support (IPS) intervention based on the Model of Human Occupation (MOHO); a prospective cohort study

**DOI:** 10.1186/s12888-020-02745-3

**Published:** 2020-07-08

**Authors:** Susan Prior, Donald Maciver, Randi W. Aas, Bonnie Kirsh, Annika Lexen, Lana van Niekerk, Linda Irvine Fitzpatrick, Kirsty Forsyth

**Affiliations:** 1 Queen Margaret University, Edinburgh, EH21 6UU UK; 2 Faculty of Health Sciences, University of Stavanger & Oslo Metropolitan University, Oslo, Norway; 3 Department of Occupational Science and Occupational Therapy, University of Toronto, Toronto, Canada; 4 Department of Health Sciences/Mental Health Activity and Participation (MAP), Lund University, Lund, Sweden; 5 Division of Occupational Therapy, University Stellenbosch, Stellenbosch, South Africa; 6 Mental Health and Wellbeing, City of Edinburgh Health and Social Care Partnership, Waverley Court, Edinburgh, EH8 8BG UK

**Keywords:** Severe mental illness, Employment, Enhanced Individual Placement & Support, Model of human occupation

## Abstract

**Background:**

Employment is good for physical and mental health, however people with severe mental illness (SMI) are often excluded from employment. Standard Individual Placement and Support (IPS) is effective in supporting around 55% of people with SMI into employment or education. Current research considers enhancements to IPS to improve outcomes for those requiring more complex interventions. Clinicians need to better understand who will benefit from these enhanced IPS interventions. This study offers a new enhanced IPS intervention and an approach to predicting who may achieve successful outcomes.

**Methods:**

This prospective cohort study included people with SMI who participated in an enhanced IPS service and had prolonged absence from employment. Secondary data analysis was conducted of data gathered in routine clinical practice. Univariate analysis coupled with previous research and clinical consultation was used to select variables to be included in the initial model, followed by a backward stepwise approach to model building for the final multiple logistic regression model with an outcome of successful or unsuccessful goal attainment (employment or education).

**Results:**

Sixty-three percent of participants in the enhanced IPS successfully attained employment or education. Significant relationships from bivariate analyses were identified between outcomes (employment or education) and seven psychosocial variables. Adapting Routines to Minimise Difficulties, Work Related Goals, and Living in an Area of Lesser Deprivation were found to be significant in predicting employment or education in the final multiple logistic regression model R^2^ = 0.16 (Hosmer-Lemeshow), 0.19 (Cox-Snell), 0.26 (Nagelkerke). Model χ^2^(7) = 41.38 *p* < .001.

**Conclusion:**

An enhanced IPS service had a 63% rate success in achieving employment or education, higher than comparable studies and provides an alternative to IPS-Lite and IPS-standard for more complex populations. Motivational and habitual psychosocial variables are helpful in predicting who may benefit from an enhanced IPS intervention supporting people after prolonged absence from employment.

**Trial registration:**

NCT04083404 Registered 05 September 2019 (retrospectively registered).

## Background

Everyone is entitled to work and fair renumeration [1] . Work is highly valued by society, offering the opportunity for self-identity and improved financial and social status [2]. However, people with severe mental illness (SMI) are often excluded from this human right [3, 4]. Standard Individual Placement and Support (IPS) (Table 1) has been identified as the most effective vocational rehabilitation intervention, with 55% of people with SMI successfully attain employment [5]. Meta-analytic studies confirm that standard IPS is superior to all non IPS interventions [6–9]. However, while effective for more than half of participants, 45% of those accessing Standard IPS do not attain their employment or education goals. Hence, recent investigations have focused on enhancements to improve outcomes for those requiring more complex interventions, to date these have primarily focused on interventions to improve cognitive and social skills [10–15]. Developing enhanced IPS and to be able to predict who secures employment or education has therefore become a priority for practice and research [11, 16, 17].

**Table 1:** Principles of Standard Individual Placement and Support & Enhanced Individual Placement and Support

Principles of Standard Individual Placement and Support (Bond et al. 2011)	
1. Eligibility is based on consumer choice	
2. Competitive employment is the goal	
3. Supported employment is integrated with treatment	
4. Personalized benefits planning is provided	
5. Rapid job search is encouraged	
6. Employment specialists build employer relationships	
7. Follow-along supports are continuous	
8. Consumer preferences are important	
Additional Principals for Enhanced Individual Placement and Support	
9. Delivered by Occupational Therapists	
10. Underpinned by the Model of Human Occupation	
11. Use of Worker Role Interview at start of program	
12. Focus on integrating daily life with working life	

Studies examining predictors of success have identified that demographic factors alone are not sufficient to predict attainment of employment goals for people with SMI [18, 19]. Clinical presentation cannot predict outcomes [20] with negative symptoms associated with unemployment [21, 22]. Some studies have found active, positive symptoms are associated with failure to attain employment [22, 23] or loss of employment [24]; while others, including a meta-analysis [15], have found no association. Psychosocial variables such as a strong work ethic [25–27], expressed desire to work [28–30], maintain habits [19, 31] and self-esteem, self-efficacy [19, 32–34] and work history [15, 35] have also been investigated and identified as predictive of success in return to employment or education.

Given the predictive nature of psychosocial variables, an enhanced IPS program (Table 1) was constructed using theoretical principles of the model of human occupation (MOHO) [36]. These include psychosocial concepts related to values and attitudes; habits and routines; performance and environment context (Table 2). The relevance of the MOHO to understanding psychosocial factors predicting return to employment from sickness absence has been established [37, 38]. A similar approach is considered appropriate for a SMI population who are unemployed and seeking employment or education [17].

**Table 2:** Theoretically defined psychosocial variables (derived from WRI)

Psychosocial areas	Theoretical Concept From Moho	Variable Derived From Theory	Definition of variable
Motivational variables	Personal Causation	Assesses abilities and Limitations;	Individual’s capacity to accurately assess his/her abilities and what they mean for work performance.
		Expectation of job success;	A client’s belief that he/she will be able to work.
		Takes responsibility;	How much responsibility a client takes for his/her work actions and their consequences.
	Values	Commitment to work;	The client’s commitment to work and importance placed on work.
		Work-related goals;	Ability to set and attain goals at his/her work setting or for finding work
	Interests	Enjoys work;	Pleasure or enjoyment client finds within work.
		Pursues interests;	The ability of an individual to assess own interests and find ways to use these skills in &/ outside of the work situation.
Habitual variables	Roles	Appraises work expectations;	Client’s ability to internalize both general and specific expectations of work.
		Influence of other roles;	How much other roles in the client’s life influence his/her return to work
	Habits	Work Habits;	Degree of organization and routine in work.
		Daily Routine;	Degree of organization and routine outside of work.
		Adapts routines to minimize difficulties;	How the person has managed use of time, routine, and habits since the injury or since being out of work.
Skills	Performance Capacity	Motor Skills	Moving body or objects in work environment
		Process Skills	Logically sequencing actions; selecting & using appropriate materials/tools; adapting performance to overcome obstacles
		Interaction and Communication Skills	
			Ability to convey intentions and needs; express self to allow for involvement and co-ordinated social action
Environment	Environment	Perception of physical work setting;	The physical environment in which the client works, or hopes to work in.
			Family’s and peers’ influence on the client’s ability to return to previous work.
		Perception of family and peers;	Influence of boss and/or company on the ability of client to return to previous work / find & keep work.
		Perception of boss and/or company;	
			Co-workers’ influence on client’s ability to return to previous work and find and keep work.
		Perception of co-workers	

This study, therefore, investigates an enhanced IPS service for people with SMI who are unemployed and seeking employment or education. It seeks to explore if theoretically derived psychosocial variables can predict employment or education outcomes in this population. While recognising the lack of consensus in defining an SMI population [39, 40] this study has adopted the definition used in IPS meta-analysis (diagnosis is predominantly psychotic disorders; disorder is persistent over time and difficulties in functioning in one or more areas of daily life) [9].

## Method

### Design

A prospective cohort study [41] was undertaken, which utilised a secondary dataset [42] . The dataset was provided by an established vocational rehabilitation service, experienced in providing a standard IPS intervention [43] with a good fidelity rating to the established IPS principles. The service simultaneously delivers a theoretically enhanced IPS service delivered by occupational therapists underpinned by the Model of Human Occupation. The study is a secondary analysis of data collected for clinical purposes. The dataset was designed in collaboration with the academic team at the inception of this service, thus ensuring that the measurement tools and data would be appropriate to research purposes [44, 45]. Data was anonymised within the clinical service and transferred using a secure email transfer. Procedures for anonymisation and transfer of data were detailed in the ethical application approved by the proportionate review sub-committee of the South Central – Berkshire B Research Ethics Committee (IRAS ID 230949).

### Measures

Clinicians use an assessment called the Worker Role Interview (WRI) [46] to identify psychosocial issues at entry into the service (Table 2) which guides the team’s interventions (Table 1). The WRI identifies psychosocial and environmental factors that influence a client’s ability to find a job, remain in work, or return to work after injury or disease [36, 46]. The WRI has a sound theoretically base, the Model of Human Occupation (MOHO) [36]. The WRI consists of a semi-structured interview, a standardised four-point rating scale is then completed by therapists. Items are summarised in Table 2 (fuller definitions are provided at clinical trials repository (NCT04083404)). The rating scale measures the impact of items on the client’s work ability [46]. The reliability and validity of the WRI has been established internationally and across populations, including SMI [47–50]. This study collapsed the 4 point WRI scale into a binary rating i.e., supports (4 = strongly supports and 3 = supports) and interferes (2 = interferes and 1 = strongly interferes). Socioeconomic status was measured by Scottish Indices of Multiple Deprivation (SIMD) which provides a relative measure of deprivation by postcode. SIMD measures: current income; employment; health; education; skills and training; housing; geographic access and crime. The Scottish Government take a national approach to calculating and weighting domains into small postcode areas, called ‘data zones’, with roughly equal population. Least deprived areas were defined as quintile 5 and more deprived areas defined as quintiles 1–4. The previous employment variable was defined as being inclusive of any work experience from teenage life to present day.

### Sample

The following inclusion criteria were applied: people with SMI, age 18 or above; consented to enhanced IPS intervention; had a goal of securing paid employment or education; had mutually agreed ongoing intervention or discharge from service prior to successful outcome. Exclusion criteria comprised: no employment/education goal; already in employment; refused engagement; withdrew from intervention. Based on the guideline of a minimum of 10 events per predictor variable [51], a sample size of 202 would allow for the robust estimation of 8 variables in the model.

### Analysis

All analysis was conducted in R data analysis software [52]. The outcome variable of interest to this study identifies those who ‘did attain’ and ‘did not attain’ their goal of employment or education programme. Logistic regression, which can handle dichotomous outcomes was therefore used in the analysis. Key assumptions of logistic regression were tested: independence of errors, linearity of relationship between continuous predictor variables and log-transformed outcome, absence of multicollinearity between predictor variables. A backward stepwise approach was adopted to building logistic regression models [53]. It is recommended that variable selection should be guided by well-established theory, clinical observations, and preliminary analysis [53]. An initial step in this study for selecting variables included bivariate analyses for determining relationships between each independent variable and the outcome. A *p*-value cut-off of 0.06 was used to guide inclusion of variables in the initial logistic model as exclusion of important variables is possible with rigid application of traditional levels (0.05) [54]. Pearson’s chi-squared test of independence using Yates correction was conducted to determine the association between categorical predictors (demographic or psychosocial) and outcome variable (obtained / did not obtain employment or education), while unpaired t tests were used to compare the difference in means of continuous predictors across the two levels of the outcome. Variables showing statistically significant associations or differences from bivariate analyses were then reviewed by clinical consultation, leading to the inclusion of seven variables in the initial logistic model. Finally, multiple logistic regression was conducted utilizing the glm function in the R stats package. The goodness of fit of models was tested using the asymptotically chi-square distributed residual deviance and pseudo R^2^. The deviance statistic along with its degrees of freedom and associated *p*-value, and three versions of R^2^ as recommended [55] are reported: the Hosmer and Lemshow R^2^, based on the chi-square score; Cox and Snells’ R^2^, based on the deviance of the model: and finally as Cox and Snells’ statistic never reaches a theoretical maximum of 1, Nagelkerke’s R^2^ provides a correction. Co-efficient estimates are reported (with standard error). Odds ratios estimating the likelihood of obtaining employment or education for different covariates are also presented. Nested models were compared using the likelihood ratio test computing a difference in deviance statistics. Final model diagnostics included checking for independence of errors, absence of multicollinearity, and lack of strongly influential outliers [53, 55, 56]. In general, models with fewer variables are simpler; and without evidence of a significant difference, the simpler model is preferred [53, 54].

## Results

### Therapists

The 21 occupational therapists who gathered data are described in Table 3, with details of their experience and numbers of cases submitted to the study. Nineteen percent did not complete certificated IPS training during their time with the service, but mentorship and supervision ensured an evidence-based approach was maintained in IPS and MOHO. IPS training was secured online through the originators of the model in the US (ipsworks.org), and more recently in the UK.

**Table 3:** Therapists’ Experience and Contribution of Data

Level of experience (all competent in MOHO and IPS)			Number of assessments included in dataset	
	n	% clinicians	n	% dataset
Novice (No IPS Certificate)	4	19	11	5%
Novice	6	29	25	12%
Experienced	8	38	96	48%
Expert	3	14	70	35%

### Participants

Referrals to the service between 2015 and 2018 were screened, this included 353 people. A total of 202 people met the inclusion criteria. Their demographic characteristics are summarised in Table 4. Reasons for exclusion from the study were: 48 did not have employment/education goal; 6 were in employment; 72 withdrew (45 due to deterioration in health and 27 refused engagement); 10 moved address; 4 deceased; 3 unavailable data; 8 other reasons. The sample was predominately male (61%) with a mean age 37.4 years (SD 10.80). Half of the sample (52%) met the diagnostic category group of ICD10 F20–29 schizophrenia, schizotypal and delusional disorders [57]. A third met the diagnostic criteria for ICD10 F30–39 Mood (affective) disorders [57]: primarily bipolar affective disorder. The group is predominately well educated, with 60% having pursued education beyond high school level. A small proportion (9%) did not attain any school-level qualifications. The majority (90%) had had some experience of paid employment.

**Table 4:** Demographic Characteristics and summary of missing data

Characteristic			Missing data	
Age	*Mean*	*SD*	0	0%
	37.4	10.8		
	n	%	n	%
Gender			0	0%
male	124	61.4%		
female	78	38.6%		
Ehnicity			0	0%
other ethnic group	17	8.4%		
White	185	91.6%		
Diagnosis			0	0%
ICD10 Schizophrenia, schizotypal and delusional disorders	105	52.0%		
ICD10 Mood [affective] disorders	67	33.2%		
ICD10 other diagnosis	30	14.9%		
SIMD			0	0%
most deprived quintile 1	38	18.8%		
quintile 2	35	17.3%		
quintile 3	33	16.3%		
quintile 4	32	15.8%		
least deprived quintile 5	64	31.7%		
Marital status			6	3.0%
Married / de facto	33	16.8%		
Single	163	83.2%		
Living Situation			4	2.0%
With others	97	49.0%		
Alone	101	51.0%		
Qualification Attainment			2	1.0%
Did Not Attain School Quals	18	9.0%		
Exams from School	61	30.5%		
Further Education	121	60.5%		
Previous Employment			0	0.0%
Previous employment	182	90.1%		
No previous experience	20	9.9%		

### Intervention

The average duration of enhanced IPS intervention to positive outcome or to discharge was just over a year: 12.2 months (SD11.40). However duration of intervention is not normally distributed and the median duration is 8.4mths (IQ range 5.1–16.7). Positive outcomes of the enhanced IPS intervention were first attainment of employment or educational opportunity, 128 participants (63%) had a positive outcome and 74 (37%) were unsuccessful.

### Bivariate analysis

Bivariate analyses on the demographic variables are presented in Table 5 and the psychosocial variables are presented in Table 6. The continuous variable age met the assumptions for unpaired t-test, and the assumptions regarding estimated distribution was confirmed for each categorical variable applying Yates correction when required.

**Table 5:** Demographic Bivariate analysis

Characteristic	Attained employment/education		Did not attain employment/education		Two Sample t-test		
	n	%	n	%	t	df	*p*
Age					−2.45	200	0.02
	n	%	n	%	Pearson’s Chi-square test. (*with Yates Correction)		
					χ^2^	df	*p*
Gender							
Male	81	40.1%	43	21.3%	0.33*	1	0.56
Female	47	23.3%	31	15.4%			
Ehnicity							
Other ethnic group	12	5.9%	5	2.5%	0.15*	1	0.70
White	116	57.4%	69	34.2%			
SMID							
Least deprived area	49	24.3%	15	7.4%	6.22*	1	0.01
More deprived area	79	39.1%	59	29.2%			
Diagnosis							
ICD10 Schizophrenia, schizotypal & delusional	62	30.7%	43	21.3%	2.97	2	0.23
ICD10 Mood [affective] disorders	48	23.8%	19	9.4%			
ICD10 other diagnosis	18	8.9%	12	5.9%			
Marital Status							
Married / defacto	24	12.2%	9	4.6%	0.95*	1	0.33
Single	101	51.5%	62	31.6%			
Living Situation							
With others	64	32.3%	33	16.7%	0.27*	1	0.60
Alone	62	31.3%	39	19.7%			
Educational Attainment							
School & Further Education Quals	119	59.5%	63	31.5%	3.86*	1	0.05
Did Not Attain School Quals	7	3.5%	11	5.5%			
PREVIOUS EMPLOYMENT							
Previous employment	117	57.9%	65	32.2%	0.33*	1	0.57
No previous experience	11	5.5%	9	4.5%			

* indicates that the Pearson's has been calculated with Yates correction

**Table 6:** Psychosocial variables Bivariate analysis

Characteristic	Attained employment/education		Did not attain employment/education		Pearson’s Chi-square test. (*with Yates Correction)		
	n	%	n	%	χ^2^	*df*	*p*
**Assesses abilities and limitations**							
**Supports**	**93**	**46.3%**	**44**	**21.9%**	3.47*	**1**	**0.06**
**Interferes**	**34**	**16.9%**	**30**	**14.9%**			
Expectation of success in work (ExpSuc)							
Supports	73	36.1%	36	17.8%	1.01*	1	0.31
Interferes	55	27.2%	38	18.81%			
**Takes responsibility (Resp)**							
**Supports**	**80**	**39.6%**	**31**	**15.4%**	7.23*	**1**	**0.01**
**Interferes**	**48**	**23.8%**	**43**	**21.3%**			
**Commitment to work (Commit)**							
**Supports**	**117**	**57.9%**	**58**	**28.7%**	5.79*	**1**	**0.02**
**Interferes**	**11**	**5.5%**	**16**	**7.9%**			
**Work related goals (Goal)**							
**Supports**	**86**	**42.8%**	**32**	**15.7%**	10.56*	**1**	**0.001**
**Interferes**	**41**	**20.4%**	**42**	**20.9%**			
Enjoys work (Enjoy)							
Supports	94	47.7%	56	28.4%	0.00*	1	1.00
Interferes	30	15.2%	17	8.6%			
Pursues interests (Interest)							
Supports	71	35.5%	38	19.0%	0.29*	1	0.59
Interferes	55	27.5%	36	18.0%			
**Appraises work expectations**							
**Supports**	**107**	**54.0%**	**51**	**25.8%**	6.14*	**1**	**0.01**
**Interferes**	**18**	**9.1%**	**22**	**11.1%**			
Influence of other roles							
Supports	97	48.0%	49	24.3%	1.69*	1	0.19
Interferes	31	15.4%	25	12.4%			
**Work habits**							
**Supports**	**92**	**46.7%**	**37**	**18.8%**	7.88*	**1**	**0.01**
**Interferes**	**34**	**17.3%**	**34**	**17.3%**			
**Daily routines**							
**Supports**	**57**	**28.4%**	**18**	**9.0%**	7.02*	**1**	**0.01**
**Interferes**	**71**	**35.3%**	**55**	**27.4%**			
**Adapts routines to minimize difficulties**							
**Supports**	**78**	**38.8%**	**22**	**11.0%**	16.43*	**1**	**0.0001**
**Interferes**	**50**	**24.9%**	**51**	**25.4%**			
Perception of physical work setting							
Supports	87	47.8%	47	25.8%	0.00*	1	0.97
Interferes	32	17.6%	16	8.8%			
Perception of family and peers							
Supports	98	52.7%	44	23.7%	1.72*	1	0.19
Interferes	25	13.4%	19	10.2%			
Perception of boss and/or company							
Supports	49	34.0%	29	20.1%	0.01*	1	0.91
Interferes	43	29.9%	23	16.0%			
Perception of co-workers							
Supports	59	43.1%	30	21.9%	0.00*	1	1.00
Interferes	32	23.4%	16	11.7%			

* indicates that the Pearson's has been calculated with Yates correction

Significant relationships were identified between employment or education and several psychosocial variables; Adapts Routines to Minimise Difficulties (*p* = 0.0001), Work Related Goals (*p* = 0.001), Appraises Work Expectations (*p* = 0.01), Work Habits (*p* = 0.01), Daily Routine (*p* = 0.01), Takes Responsibility (*p* = 0.01), Least Deprived area (*p* = 0.01), Commitment to Work (*p* = 0.02), age (*p* = 0.02). Borderline significant relationships were identified between Outcome and Educational Attainment (*p* = 0.05) and Assesses Abilities and Limitations (*p* = 0.06). Following clinical consultation, two variables i.e., Work Habits and Assesses Abilities and Limitations, were excluded from the initial logistic model. Both variables were considered challenging to rate in clinical practice for long term unemployed and, therefore, of limited clinical applicability in this population.

### Multiple logistic regression

The exclusion of cases containing missing data in the covariates of interest resulted in an inclusion of 195 cases in the multiple logistic regression [58]. One hundred and twenty four (64%) had a successful outcome and 71 (36%) were unsuccessful. Multiple logistic regression models were built iteratively with non-significant variables removed at each step and the resulting nested model compared with the fuller model to assess significant difference in fit, three statistically significant variables are included in the final model: Adapting Routines to Minimise Difficulties, Work Related Goals, and Living in an Area of Lesser Deprivation. The variable Age has borderline significance within the model. Independence of errors was confirmed: DWT statistic = 1.93 *p* > 0.05. Multicollinearity was excluded, average VIF = 1.2 with a range from 0.74–1.35; outliers were identified, three cases (1%) were identified as outliers to the final model. A Cook’s influence level of only 0.03 in all cases demonstrated the outliers have little influence over the final model. Therefore these cases were included. The final model is presented in Table 7. A rating of ‘supports’ in either Adapting Routines to Minimise Difficulties, or Work Related Goals will increase the odds of obtaining employment or educational. A ‘supports’ rating in Adapting Routines to Minimise Difficulties increases the likelihood of goal attainment by 261%,. A ‘supports’ score on Work related Goal increase the likelihood of obtaining employment or education by 143%. Living in a Less Deprived Area improves the odds of attaining employment or education by 166%. Age while not reaching levels of statistical significance within the model (*p* < 0.1) a younger age increases the likelihood of a successful outcome.

**Table 7:** Model of attainment of employment or education for enhanced IPS

	*b (SE)*	*95% CI for Odds Ratio*		
		*Lower*	*Odds Ratio*	*Upper*
Constant	−0.03 (0.72)	0.23	0.96	3.91
Adapts Routines to Minimize Difficulties	1.28 (0.39) ***	1.72	3.61	7.86
Work-Related Goals	0.89 (0.36)*	1.21	2.43	4.99
Appraises Abilities and Limitations	0.37(0.42)	0.63	1.45	3.34
Work Routines	0.06 (0.41)	0.47	1.07	2.38
Responsibility	0.09 (0.39)	0.51	1.09	2.33
Age	−0.03 (0.02)	0.94	0.97	1.00
Living in Least Deprived Area	−0.98 (0.39) *	1.25	2.65	5.90

*R*^*2*^ *=* 0.16 (Hosmer-Lemeshow), 0.19(Cox-Snell), 0.26 (Nagelkerke)

Model 흌^2^(7) = 41.38 *p < .001*

*p < 0.05 ***p < 0.001

The datasets analysed during the current study are available in the clinical trials repository (NCT04083404).

## Discussion

This study investigated an enhanced IPS service for people with SMI who are unemployed and seeking employment or education and explored if theoretically derived psychosocial variables can predict employment or education outcomes in this population.

### Outcomes of enhanced IPS

Participants in this enhanced IPS programme had a 63% successful attainment of employment or education, which compares favourably to other UK non-experimental studies of standard IPS. An early study comparing standard IPS with traditional vocational rehabilitation (TVR) reported 56% of participants in standard IPS attained employment or education compared to 22% in TVR [59]. The same authors conducted a further study investigating standard IPS for young people with first episode psychosis [60] . Over an 18 month period the employment or education rates improved by 43% (baseline = 38%; 18mths = 81). A later study focused on early intervention services, which included a 12 month trial of standard IPS, found employment/education attainment improved by 23% (baseline rate = 23%; 12mth rate = 35%) [61]. It could, therefore, be concluded that the enhanced IPS program in the present study, with a success rate of 63%. Caution should be exercised in comparing this present study to the 55% success rate of international randomised controlled trials [5], due to the different research designs used. In addition, the UK randomised control trials (RCT) of standard IPS outcomes are 46% [62] and 22% [63]. Previous research has offered an explanation for the lower UK outcomes compared with US studies are differences in employment protection legislation availability of welfare/disability benefits [64, 65].

Results of this present study should also be considered in the context of other studies investigating enhancements of IPS. To date the only other adaptation to standard IPS studied in the UK is the IPS-Lite [62], establishing that a time-limited (up to 9 months) implementation of IPS-Lite yielded a success rate of 41% compared to standard IPS employment rate of 46%. The authors argue that the time limit increased capacity of IPS services and focuses efforts on those most likely to benefit. The present study compliments IPS Lite by offering an enhanced IPS to support those unable to attain employment within the IPS-Lite defined 9 months, particularly those who require a more complex intervention than standard IPS. Meta-analytic study of international RCTs found enhanced IPS was more effective than supported employment (RR 1.40, 95% CI 0.92 to 2.14) [9]. Individual placement and support enhanced with social skills training [66], found 82.8% of participants in the enhanced IPS attained employment compared to 61.5% of standard IPS participants. However, authors acknowledge that the study sample is small and those with more severe symptoms were excluded. A study of IPS enhancement through cognitive remediation [67] identified enhanced IPS (69.6%) over standard IPS (14.3%), but, low adherence to IPS fidelity was reported in both arms of the study, which may explain the poor standard IPS outcome. Moreover, another study investigating enhancement through cognitive remediation reported enhanced IPS yielding 15.2% employment outcomes and 14.9% for standard IPS [14]. It could be concluded that alternative enhancements are still in development and reliable research findings not yet established. The present study offers an additional enhanced IPS program not currently being studied within the field.

### Psychosocial variables predict outcomes

Previous studies have argued that future research should investigate psychosocial variables which may predict success [17]. Our study found seven psychosocial items from the intake assessment were significantly correlated with outcome (*p* < 0.01), and two of these items (Adapts Routines to Minimise Difficulties and Work Related Goals) contribute to a multiple regression model. It could, therefore, be argued that an assessment (e.g., WRI) of psychosocial factors at commencement of an enhanced IPS intervention would be valuable. Developers of IPS recommend that vocational assessments are not required in IPS programmes [68], although do encourage information gathering through the career profile interview. Reservations regarding assessments seem to be primarily founded on the perception that vocational evaluation is limited to performance capacity measurement, and will lead to the screening out of individuals with limitations [68]. This study has provided evidence of the value of the Worker Role Interview which identifies psychosocial strengths which may be built upon. Clinicians assessing psychosocial strengths, using the WRI, at the beginning of an enhanced IPS intervention would allow appropriate targeting of enhanced IPS interventions, thus improving efficiencies [11, 17].

Previous research has also found work related goals expressed as a desire to work [29, 30] is predictive of positive employment outcome. Work Habits and Routines are also emerging as promising predictors of employment or educational outcomes. Work Routines was also significant in a study predicting return to work from sickness absence [38] at both univariate level and within multivariate regression. Adapts Routines to Minimize Difficulties was also significant at univariate level in return to work from sickness absence study [38] and has also been found to differentiate between employed and unemployed US veterans [69]. Work Habits, while significant in the present study, has not been found to be significant in any other study predicting employment outcomes. Perception of Family and Peers, was not found to be significant, however, was considered a clinically important factor by therapists in this present study. Non significance maybe due to missing data (8%) [55], resulting from the difficulty rating the variable. Additionally, therapists involved in this present study concurred with other authors [34, 70, 71] that the environmental factors are clinically important. The final multiple logistic regression model included Adapts Routines to Minimize Difficulties and Work-related Goals. Significant finding of Work-related Goals has resonance with previous research [29, 30] and a rating of “supports” requires the individual to not only express a desire for employment but for this to be demonstrated in behaviour “setting realistic goals and setting clear plans for finding work” [46]. Similarly the Adapts Routines to Minimize Difficulties item is behavioural in nature and is defined as “how the person has managed use of time, routine, and habits since the injury or since being out of work” [46]. Together these items offer measurable rating of the enactment of desire for employment. The present study relied upon routine collected clinical data and therefore only captured attainment of employment or education. It is recommended that future studies should investigate if psychosocial variables predict retention of employment and education by including greater detail on the type of work or educational programme secured and the duration.

### Population served

This present study was focused on a population who are unemployed and often have minimal or distant employment experience. While four items overlapped by being significant across sickness absence population and a prolonged absence from employment population at univariate level (Adapts Routines; Daily Routines; Takes Responsibility and Commitment to Work), the multiple regression model identified different items as being significant in each population. This difference perhaps reflects how psychosocial variables vary depending on people having an employment experience to reflect upon and how daily routines change to reflect a short period of absence compared to a complete non-existence of worker role. Therapists described difficulty rating some of the WRI items due to the absence of recent employment experience to reflect upon and this resulted in missing data. This was particularly evident in all four environmental items. Similar challenges were acknowledged in a previous study [50]. Therefore practitioners need to be creative in considering interventions which allow clients to have a real work experience prior to rating the WRI in populations who have been out of work for a longer time period.

### Limitations

The demographic profile of this present study is similar to other IPS studies [63, 72–76] in that it does not represent the SMI population. The present study had a high rate of those in marital/equivalent relationships; more males than females and very few younger people. While it is recognised that young people should have early access to IPS programmes it is notable that the mean age of many IPS RCTs is over 35 yrs. [63, 72–76]. Age was a significant variable at univariate analysis in this present study but was not significant in the multiple regression model. Better representation of young people in this sample would perhaps have increased significance. This study had an unusually high (90%) previous employment rate. A history of employment is recognised as predicting success [35, 77]. However, recency is relevant to the predictive nature of employment history [19, 78]. Studies in the field usually define this variable as a reported work history in the last 3–5 years. The high percentage of employment history identified in this study is perhaps explained by the over-inclusive definition of previous employment being any work including from teenage life, which does not attend to the recency of employment. Therefore comparisons between this present study and others is not possible. In the present study 17% of the sample were married or de facto relationship, while in UK population study only 0.5% of those with a psychotic illness were in a similar relationship [79]. In this study 51% were living alone, compared with a national average for people with SMI of 35% [80]. The sample was also predominately well educated, with 60% having pursued education beyond school level, had a low ethnic diversity and a third (32%) of the sample lived in an area classified as in least deprived. Future studies need to be clear about the population who received the IPS service to allow for a clearer understand of which subset of the SMI population is being served. It is possible that the patients excluded from the study may have skewed data however more than half of those excluded were not appropriate participants in an IPS programme not having an employment goal, being in employment or refusing to intervention. Withdrawal from intervention is also a widely recognised challenge in this population [81].

## Conclusion

The study adds to the evidence base of vocational rehabilitation introducing a new enhanced IPS intervention based on the MOHO which achieved 63% return to employment or education and can provide alternatives to standard IPS and IPS-Lite for people who have more complex needs. This study also extends knowledge regarding the relevance of the WRI with an SMI population. Motivational and habitual psychosocial variables are identified as helpful in predicting who may benefit from an enhanced IPS intervention after prolonged absence of previous employment. This study also provides an example of embedding research into core health services promoting sustainability. The challenge of sustainability is a factor to consider in RCT research, where well-funded and managed trials are successful for the duration of implementation but are not integrated into mainstream services [82–84].

## Data Availability

The datasets analysed during the current study are available in the clinical trials repository (NCT04083404).

## Abbreviations

CI: Confidence Interval
DWT: Durbin-Watson Test
ICD10: International Classification of Diseases, Tenth Revision
IPS: Individual placement and support
IQ: Interquartile
IRAS: Integrated Research Application System
MOHO: Model of human occupation
R^2^: Coefficient of determination
RCT: Randomised control trials
SD: Standard deviation
SIMD: Scottish Indices of Multiple Deprivation
SMI: Severe mental illness
UK: United Kingdom
US: United States of America
VIF: Variance Inflation Factor
WRI: Work Role Interview
